# Supplementary material for: Equity incentive contract characteristics and company operational performance—An empirical study of Chinese listed companies
Source: PLoS One. 2023 Feb 6;18(2):e0281244. doi: 10.1371/journal.pone.0281244 (PMC9901763; doi:10.1371/journal.pone.0281244)
Supplement: Attachment — Submitted filename: Response to Reviewers.docx [file pone.0281244.s001.docx]

**Suggestions of Reviewer 1**

**Dear expert, we are very grateful for your valuable suggestions, and we will make a specific reply below.**

1. Authors should use Plos One style in writing "abstract"; please check the author's instruction.

***Response:*** *As suggested, the summary section has been tracked, Please refer to* ***line*** *13-37 in tracked version.（all “****line****” refers to tracked version）*

*“Equity incentive, as an institutional arrangement for coordinating the interests of shareholders and managers, has been widely implemented by public companies in developed capital markets in Europe and America. However, does it work and/or when might it be more effective in emerging market economies like China? To understand the effects of equity incentive plans implemented by listed companies in China and the potential influence of the general characteristics of contracts on the effectiveness of equity incentive plans. Based on* *behavioral decision theory, this study adopts a multivariate linear regression model to analyze the 1695 equity incentive plans implemented in Chinese listed companies between 2010 and 2018 with their two-year lagged performance data.* *The empirical results show that the operational performance of companies after implementing equity incentive plans shows the trend of polarization. In the 95% confidence interval, the effect of restrictive stock incentive and exercise-constrained variables is not significant, while the validity period has a significant positive correlation and incentive intensity has a significantly negative correlation with the company’s operational performance. Furthermore, the negative effects mentioned above become more obvious with a longer plan implementation period. Based on these conclusions, we suggest that companies could adopt equity incentive plans with a relatively longer validity period and more reasonable incentive intensity. Additionally, it would be better for companies to select non-restricted stocks as incentive tools if there is no obvious preference.”*

2. No need to have the results in the introduction section.

3. Authors should add the “research structure” for the whole manuscript in the introduction section.

***Response to 2 and 3:*** *The research conclusion has been deleted in the introduction and the structure of the paper has been added，Please refer to* ***line*** *96-104 in tracked version.*

*“The rest of the paper is arranged as follows: the second part is the review of relevant literature, including the motivation of equity incentive, equity incentive scheme and equity incentive effect; The third part is the proposal of research hypotheses, including the causal effects between equity incentive media and corporate performance, the validity period of equity incentive and corporate performance, and the intensity of equity incentive and corporate performance. The fourth part is empirical research, including research design, descriptive statistics and regression analysis. The fifth part is the discussion and conclusion, including the research conclusion, practical and theoretical significance and future research direction.”*

4. Both Introduction and LR parts need to be improved and enriched with extra new papers in the field.

13. Regarding references: most of the references are old. This topic is considered almost as new topic, and many important articles have been published recently in this field. I advise you to refer to previously published articles in Plos One and other top journals to include more updated papers in your study.

***Response to 4 and 13:*** *We make some complement，Please refer to* ***line*** *70-74 ,* *Line175-176, line189-190, line532-535 and so on in tracked version.*

***line*** *70-74“For example, study finds that equity incentives are negatively correlated to stock price crash risk in the golden hand cuff regions while equity incentives are positively correlated to stock price crash risk in the golden watch region [3]. In addition, research reveal that high equity incentives could strength CEOs’ degree of earnings management activities [4]”*

*Line175-176”* *For example, study reveal that equity incentive exerts positive influence on enterprise’s innovation [26].”*

*line189-190,”* *What’s more, study revealed that equity incentive could exert significant influence on corporate innovation performance [30]”*

*line532-535”* *Previous studies mostly discussed the economic consequences from the single variables such as the mode, validity period and intensity of equity incentive. For example, research only select the percentage of shares held by managers as the proxy of equity incentive [26].”*

5. Authors need to position their article compared to the academic literature and specify the gap they are trying to fill.

***Response:*** *We add new articles to strength this aspect, for example,* *line70-74,* *line154-155, Line175-176,* *line189-190 and so on in tracked version.*

*line70-74 “For example, study finds that equity incentives are negatively correlated to stock price crash risk in the golden hand cuff regions while equity incentives are positively correlated to stock price crash risk in the golden watch region [3]. In addition, research reveal that high equity incentives could strength CEOs’ degree of earnings management activities [4].”*

***line*** *154-155, “Meanwhile, research compared the different effectives among some incentive conditions [20]”*

***line*** *175-176, “For example, study reveal that equity incentive exerts positive influence on enterprise’s innovation [26]”*

*line189-190,* “*What’s more, study revealed that equity incentive could exert significant influence on corporate innovation performance [30]”*

6. The contribution of this paper and the importance of this contribution are not clear; authors should state their contribution and show the importance of this contribution.

8. The authors are not comparing their results with previous work. They are not referring to any reference.

9. The conclusion should be written in paragraphs, not as points.

10. The results and analysis section; it only contains the results with a little discussion. More discussions are needed. Also, the authors should explain more precisely the effect of their results on the Chinese market. In other words, authors should interpret the results beyond their statistical significance to demonstrate their economic impact.

11. Authors should include the "suggestion" section in the conclusion

12. Authors should add the "practical and theoretical implication" and the "limitations and further studies" sections at the end of their paper.

***Response to 6,8,9,10,11 and 12:*** *According to the advices, We make some complement，for example, comparing with previous work and explaining the economic impact in* ***line*** *488-491,* ***line*** *504-509; We redesign the “conclusion”, in the tracked version, we add Theoretical contribution and practical implications in* ***line*** *530-560, suggestions in* ***line*** *561-574, Further Research Directions in* ***line*** *575-584 and so on in tracked version.*

***line*** *488-491, “This conclusion is not only different from extant study that "stock option has a more obvious effect on executive incentive"[40], but also different from research that "equity incentive will increase executive speculation and weaken enterprise value"[2].”*

***line*** *502-507, “The research conclusion is consistent with the views of some previous study [40], and is also close to the research results on American enterprises [49]. However, it is just the opposite of study that found a longer term of validity will induce excessive short-term investment behavior, which may be related to the term of the manager [50]. If the manager faces job changes in the short term, the longer term of validity incentive will increase his agency behavior.”*

***line*** *528-557, “5.2. Theoretical contribution and practical implications*

*The marginal theoretical contribution of the paper may be reflected in the following aspects: firstly, the paper enriches the relevant research framework of equity incentive, Previous studies mostly discussed the economic consequences from the single variables such as the mode, validity period and intensity of equity incentive. For example, research only select the percentage of shares held by managers as the proxy of equity incentive [26]. This paper makes a comprehensive study on the above three factors of equity incentive and uses the panel data of Shanghai and Shenzhen listed companies for regression test, so as to obtain more prudent and detailed research conclusions, It also adds more intuitive empirical evidence to verify whether equity incentive intensifying or alleviating the agency phenomenon.*

*Secondly, this paper broadens the research on the influencing factors of enterprise performance. In the past, many literatures have discussed the impact of enterprise financial leverage [47]、enterprise risk management [46]、corporate social responsibility [56]、Confucian culture [57]、enterprise power structure [58] and other factors on corporate performance，This paper explores the impact of equity incentive on corporate value and expands the research on the pre variables of enterprise performance.*

*At the same time, this paper may also have some practical implication, the research conclusion shows that the existing equity incentive model has no significant impact on corporate performance, which shows that in the practice of enterprise management, we should strengthen the innovation of incentive model and explore the equity incentive method suitable for Chinese local enterprises, so as to enhance the practical value of the strategy. In addition, the study found that the longer the validity period of equity incentive, the more it can promote enterprise performance. This conclusion shows that the validity period can be appropriately increased when designing the duration of equity incentive in practice, which helps to maintain the convergence of the interests of management and enterprises. Finally, the study found that higher equity incentive intensity is not conducive to enterprise performance. This conclusion shows that managers holding higher equity share may provide convenience for their self-interest behavior and further aggravate the occurrence of agency phenomenon. Therefore, in practice, we should determine the reasonable range of equity incentive intensity and carefully use the incentive with too high intensity.”*

***line*** *561-574, “5.23. Suggestions. The design of incentive mechanism in team is a very important issue, which is related to organizational performance [59]. Based on the research results listed above, this paper offers the following suggestions on the equity incentive plans of listed companies. First, subject matter shows an unapparent negative correlation with company operational performance. Therefore, enterprises should consider their actual situation first when choosing the incentive medium. If there is no preference for any subject matter, companies could consider non-restricted stocks first. Second, enterprises should create reasonable incentive schemes based on their current situation. Based on the significant effect of the validity period and incentive intensity, enterprises can choose a long incentive validity period and they should be more cautious when selecting the intensity of motivation. Last but not the least, equity incentive plans can be an efficient way to test the managerial power of a company. Companies with a “Welfare plan" should pay more attention to improving the internal management structure and reducing management power.”*

***line*** *575-584, “5.4. Further Research Directions. This paper discusses the relationship between the types of equity incentive and corporate performance, and it is found that there is no obvious linear relationship between them, In the future research, we can further explore the possible U-shaped and other nonlinear relationship between them. In addition, this paper verifies the significant impact of the validity period and intensity of equity incentive on corporate performance, but the mechanism and boundary of equity incentive have not been discussed in depth, In the future research, we can further analyze the path mechanism of both the impact of equity incentive validity and incentive intensity on corporate performance, as well as the boundary effects such as action intensity in different situations from the perspective of theory and demonstration.”*

14. Editing for English is required

***Response:*** *owing to the tracked version is too chaotic, meanwhile the paper may confront new change, hence, we’d like to edit the English in the following version.*

15. The author mentioned the name of the researchers in addition to the number of the reference in many pages. As in rows of 137, 141, 161, 172, 179, etc

***Response:*** *According to the advice, we tracked the above questions, for example,* ***line*** *152-155 and so on, “Homogeneously, study reached a similar conclusion by discussing small-scale companies, pointing out that a restricted stock mode could weaken agency behavior and reduce agency costs[19]. Meanwhile, research compared the different effectives among some incentive conditions [20].”*

7. Methodology: the data used in this research are from 2010 till 2014; these data are outdated. Updated data are required.

***Response:*** *According to the advice, new data in paper is from 2010 to 2018, please refer to* ***line*** *299-307, “implemented in Shanghai and Shenzhen A-share markets between January 1, 2010 and December 31, 2018. Thus, we obtained 1737 equity incentive cases.*

*In order to ensure the accuracy and validity of the research results further, we further screened the samples and selected them in accordance with the following principles:*

*1）Because the different business content of finance and insurance industries leads to different compositions of company assets, liabilities, and owner’s equity, they have an independent accounting system. Therefore, this study excluded financial and insurance companies.*

*2）This study excluded invalid values that were missing significant data.*

*Following the above steps, 1695 valid samples were obtained.”*

*And the following statistics tables. For example, table 1 in* ***line*** *344, table 2 in* ***line*** *349, ……table 5 in* ***line*** *467.*

**Reviewer 2**

**Dear expert, we are very grateful for your valuable suggestions, and we will make a specific reply below**

1.The structure of the paper should be presented in the introduction.

***Response:*** *The structure of the paper has been added，Please refer to* ***line*** *96-104 in tracked version**（all “****line****” refers to tracked version）. “The rest of the paper is arranged as follows: the second part is the review of relevant literature, including the motivation of equity incentive, equity incentive scheme and equity incentive effect; The third part is the proposal of research hypotheses, including the causal effects between equity incentive media and corporate performance, the validity period of equity incentive and corporate performance, and the intensity of equity incentive and corporate performance. The fourth part is empirical research, including research design, descriptive statistics and regression analysis. The fifth part is the discussion and conclusion, including the research conclusion, practical and theoretical significance and future research direction.”*

2. The authors should explain why China is worth studying in relation to other countries.

***Response:*** *We make some complement, for example, adding some Chinese study in order to elaborate the complexity of equity incentives in Chinese listed companies, please refer to* ***line*** *70-75, “For example, study finds that equity incentives are negatively correlated to stock price crash risk in the golden hand cuff regions while equity incentives are positively correlated to stock price crash risk in the golden watch region [3]. In addition, research reveal that high equity incentives could strength CEOs’ degree of earnings management activities [4].”.* ***line*** *87-92, “Compared with developed capital markets such as Europe, America and Japan, China's equity incentive system is implemented late, the system design is not perfect, and the equity incentive schemes implemented by listed companies are mixed. Therefore, exploring the stock incentive of Chinese listed companies will help to further clarify the economic effect of equity incentive, so as to provide more specific reference value for emerging market countries.”.* ***line*** *175-176 “For example, study reveal that equity incentive exerts positive influence on enterprise’s innovation [26].” in tracked version.*

3. Tobin Q has been used extensively by papers focused on emerging capital markets. I strongly recommend you to use this measure for robustness checks.

***Response:*** *According to the advice, We make a supplement in “Robustness Check” section in* ***line*** *435-467. “To investigate the robustness of the evaluation methods and conclusions further, this study chose Tobin QROE as an alternative variable to evaluate the operational performance of a company. Similarly, this study constructed the following multivariate linear regression models………”, and the following part*

4. You should clarify the contributions of the paper which are not elaborated well in the current paper. You can talk about the following contributions: What insights can you provide based on your finding? Do they push forward our understanding? What should we do with your research? Do you have any suggestions to improve the current regulation or practice? Adding the above discussion and extending your literature review may help you make more contributions and position your contributions better.

The authors should highlight the limits of their research.

***Response:*** *According to the advices, We make some complement，for example, comparing with previous work and explaining the economic impact in* ***line*** *488-491,* ***line*** *504-509; We redesign the “conclusion”, in the tracked version, we add Theoretical contribution and practical implications in* ***line*** *530-560, suggestions in* ***line*** *561-574, Further Research Directions in* ***line*** *575-584 and so on in tracked version.*

***line*** *488-491, “This conclusion is not only different from extant study that "stock option has a more obvious effect on executive incentive"[40], but also different from research that "equity incentive will increase executive speculation and weaken enterprise value"[2].”*

***line*** *502-507, “The research conclusion is consistent with the views of some previous study [40], and is also close to the research results on American enterprises [49]. However, it is just the opposite of study that found a longer term of validity will induce excessive short-term investment behavior, which may be related to the term of the manager [50]. If the manager faces job changes in the short term, the longer term of validity incentive will increase his agency behavior.”*

***line*** *528-557, “5.2. Theoretical contribution and practical implications*

*The marginal theoretical contribution of the paper may be reflected in the following aspects: firstly, the paper enriches the relevant research framework of equity incentive, Previous studies mostly discussed the economic consequences from the single variables such as the mode, validity period and intensity of equity incentive. For example, research only select the percentage of shares held by managers as the proxy of equity incentive [26]. This paper makes a comprehensive study on the above three factors of equity incentive and uses the panel data of Shanghai and Shenzhen listed companies for regression test, so as to obtain more prudent and detailed research conclusions, It also adds more intuitive empirical evidence to verify whether equity incentive intensifying or alleviating the agency phenomenon.*

*Secondly, this paper broadens the research on the influencing factors of enterprise performance. In the past, many literatures have discussed the impact of enterprise financial leverage [47]、enterprise risk management [46]、corporate social responsibility [56]、Confucian culture [57]、enterprise power structure [58] and other factors on corporate performance，This paper explores the impact of equity incentive on corporate value and expands the research on the pre variables of enterprise performance.*

*At the same time, this paper may also have some practical implication, the research conclusion shows that the existing equity incentive model has no significant impact on corporate performance, which shows that in the practice of enterprise management, we should strengthen the innovation of incentive model and explore the equity incentive method suitable for Chinese local enterprises, so as to enhance the practical value of the strategy. In addition, the study found that the longer the validity period of equity incentive, the more it can promote enterprise performance. This conclusion shows that the validity period can be appropriately increased when designing the duration of equity incentive in practice, which helps to maintain the convergence of the interests of management and enterprises. Finally, the study found that higher equity incentive intensity is not conducive to enterprise performance. This conclusion shows that managers holding higher equity share may provide convenience for their self-interest behavior and further aggravate the occurrence of agency phenomenon. Therefore, in practice, we should determine the reasonable range of equity incentive intensity and carefully use the incentive with too high intensity.”*

***line*** *561-574, “5.23. Suggestions. The design of incentive mechanism in team is a very important issue, which is related to organizational performance [59]. Based on the research results listed above, this paper offers the following suggestions on the equity incentive plans of listed companies. First, subject matter shows an unapparent negative correlation with company operational performance. Therefore, enterprises should consider their actual situation first when choosing the incentive medium. If there is no preference for any subject matter, companies could consider non-restricted stocks first. Second, enterprises should create reasonable incentive schemes based on their current situation. Based on the significant effect of the validity period and incentive intensity, enterprises can choose a long incentive validity period and they should be more cautious when selecting the intensity of motivation. Last but not the least, equity incentive plans can be an efficient way to test the managerial power of a company. Companies with a “Welfare plan" should pay more attention to improving the internal management structure and reducing management power.”*

***line*** *575-584, “5.4. Further Research Directions. This paper discusses the relationship between the types of equity incentive and corporate performance, and it is found that there is no obvious linear relationship between them, In the future research, we can further explore the possible U-shaped and other nonlinear relationship between them. In addition, this paper verifies the significant impact of the validity period and intensity of equity incentive on corporate performance, but the mechanism and boundary of equity incentive have not been discussed in depth, In the future research, we can further analyze the path mechanism of both the impact of equity incentive validity and incentive intensity on corporate performance, as well as the boundary effects such as action intensity in different situations from the perspective of theory and demonstration.”*

***In the end, We thank you very much for your valuable advice, and make above-mentioned modification, we add the two papers (Anton,2018; Anton,2019) in reference 46 and 47.***
