# Supplementary material for: Equity incentive contract characteristics and company operational performance—An empirical study of Chinese listed companies
Source: PLoS One. 2023 Feb 6;18(2):e0281244. doi: 10.1371/journal.pone.0281244 (PMC9901763; doi:10.1371/journal.pone.0281244)
Supplement: Attachment — Submitted filename: Referee_report_PONE-D-22-03250_R2_.docx [file pone.0281244.s002.docx]

Dear Author(s)

I would like to thank the author(s) for your submission and appreciate the opportunity to read and review your manuscript. I enjoyed reading it. This manuscript is relevant to contemporary finance research

My comments concern only with clarity, and text. I would recommend publication, after the issues below are addressed.

Changes which must be made before publication:

Major changes:

1. Abstract: : It's okay
2. The Keywords: the Keywords section should deserve more attention, 2-3 keywords should be added.
3. The introduction section: I think it is okay but needs more attention from the author(s).The introduction should give a short summary of your study. The introduction should tell the reader why the study is important, and should seek the attention of the reader by stimulating interest, desire and action).
4. The literature review section: It's okay.
5. Methodology/Research methods: Methodology/Research methods section is missing. Please add a paragraph about the methodology used before the research design.
6. The results section: should be better analyzed and developed further.
7. Conclusions and Further Directions: please augment its quality with more depth, rigor and substance. I recommend you to rewrite this section from p. 27 to p. 31 and avoid use the numeric style in writing this section. This style may be more suitable with reports.
8. The language of the manuscript: the language of the manuscript needs a "Careful Editing" by a native speaker (academic writing style should deserve more attention).

Additional comments: the author(s) are recommended to do the following to increase the manuscript readability.

1. To start with, the manuscript should more clearly and more explicitly spell out its objectives.
2. Please keep your writing in a scientific writing style, the most effective scientific writing aims for accuracy, clarity and objectivity.
3. Please be careful in using the abbreviations, abbreviated words, and definitions throughout the manuscript. Please see p. 16 table 1 the author(s) defined GROW as follows:
   - (Profit of this year – Profit of last year) / Profit of last year

I think "GROW" can be defined as follows:

- Net profit growth (%) =  (current period NP-Prior Period NP)/Prior Period NP

4. Please be careful in using "Punctuating", e.g. (comma, full stop, etc…) throughout the paper.
5. Some of the terminology used in the manuscript needs to be unified. For instance, the use of 4 different words that have the same meaning interchangeably could confuse some readers such as "paper/ article/research/study".

Finally, based on the above-mentioned comments, I suggest “major revision” of the manuscript before publication.
