# Supplementary material for: Equity incentive contract characteristics and company operational performance—An empirical study of Chinese listed companies
Source: PLoS One. 2023 Feb 6;18(2):e0281244. doi: 10.1371/journal.pone.0281244 (PMC9901763; doi:10.1371/journal.pone.0281244)
Supplement: Attachment — Submitted filename: Response to Reviewers.docx [file pone.0281244.s003.docx]

**Dear Editor and Reviewers,**

**We are very grateful for your valuable suggestions, and we will make a specific reply below.**

**1. Abstract: It's okay**

**2. The Keywords: the Keywords section should deserve more attention, 2-3 keywords should be added.**

**Response:** As suggested, we add some keywords *“Keywords: equity incentive, operational performance, Empirical research, Chinese listed companies, Ownership concentration”* in line 30 -31 in tracked version （line 25 in English edited version）. （all “line” refers to tracked version, and “line” in brackets refers to English edited tracked version.）

**3. The introduction section: I think it is okay but needs more attention from the author(s). The introduction should** **give a short summary of your study. The introduction should tell the reader why the study is important, and should seek** **the attention of the reader by stimulating interest, desire and action).**

**Response:** As suggested, we have made following changes:

(1) Added the manuscript *“As an emerging economy with immature capital market and weak corporate governance, how listed companies to design their equity incentive plans would be more helpful to improving operational performance? Answering this question Therefore, exploring the stock incentive of Chinese listed companies will help to further clarify the economic effect of equity incentive”* in lines 82-84 ( line 83-87);

(2) Revised *“The results of data analysis of 1695 equity incentive cases of China’s listed companies indicate that equity incentive plans are more popular in high-tech industries. Validity periods of incentive plans have significant postive impact on firms’ operational performance, while incentive intensity has a significant negative effect on firms’ operation performance. However, exercise constraints of incentive plans have no significant impact on firms’ operational performance. Our findings have enriched the literature of equity incentive and provide some practical implication for the design of equity incentive plans.”* in lines 90-97 (lines 88-96) in order to give a short summary of our study and seek the attention of the reader.

**4.The literature review section: It's okay.**

**5.Methodology/Research methods: Methodology/Research methods section is missing. Please add a paragraph about the methodology used before the research design.**

**Response:** Following this suggestion, we added a new section “Methodology” line 271-261 (line 259-348), and made following modifications:

(1) Move “4.1 research design” to “Methodology” section ( line 290-346 in former edition of manuscript), as subsection “4.1. Sample and sources of data” in line 275-292 (lin 266-282) and subsection “4.2. Variable definition” in line 293-318 (283-319);

(2) Move model specification part in “4.2.2. Results of Multiple linear regressions” ( line 364-384 in former edition of manuscript) to “Methodology” section as a subsection “4.3. Model specification”, in line 329-367 ( line 320-354);

(3) All contents mentioned above were revised to fit to the new section.

**6. The results section: should be better analyzed and developed further.**

**Response:** Following the suggestion, we have revised the results section to make it more accurate and academic style. Major changes are as follows:

(1) “*A comparison of the three-year ROA data after implementation shows that the mean value of ROA is declining, but the max value is increasing, and it shows a growing polarization trend in the three years. Therefore, the operational performance of listed companies who have implemented equity incentive plans experienced large-scale variance, which means that the impact of stock incentive plans with different characteristics might be different.*” In line 374-380 ( line 361-365);

(2) “*Pearson correlation coefficients for each variable are presented in Table 3. It is worth noting that there was a negative correlation between STOCK and the other variables. Because STOCK is a virtual variable and 1 represents restricted stocks while 0 represents non-restricted stocks, it can be found that restricted stock has an inhibitory effect on other explanatory and control variables. By comparing all Pearson correlation indices between variables, it can be found that all value of correlation coefficients are below 0.4 (a few indices are around 0.4). Hence, the correlations between these variables are weak. Therefore, there should have no multiple co-linear problems between these variables in the regression models.*” In line 405-415 ( line 374-381);

(3) “From the results of the regression model shown above, it can be seen that the adjusted coefficients of determination are above 0.25, which means that these models have strong explanatory ability. A comparison of the last four models (Models (6)–(8)) shows that Model (6)’s goodness of fit is the best, Model (7) is the second best, and Model (8) is the worst. The results shows that all the regression models are significant at the 0.001 level.

…

The above results indicate that, an equity incentive plan with a long validity period can greatly promote a company’s operational performance, and the impact reaches its peak in the second year after equity incentive implementation. However, incentive intensity has an negative effect on a company’s operational performance.” in line 425-468 ( line 390-427).

**7.Conclusions and Further Directions: please augment its quality with more depth, rigor and substance. I recommend you to rewrite this section from p. 27 to p. 31 and avoid use the** **numeric style in writing this section. This style may be more suitable with reports.**

**Response:** As suggested, we have dropped the numeric style and made some supplement, such as:

*“Undoubtedly, senior executives of high-tech enterprises usually have higher professional skills, and such professional skills have a higher threshold. The above factors determine that it is necessary to implement appropriate incentives.”* in line 507-509 ( line 448-450);

*“they used China's GEM listed companies as a sample, this kind of listed companies started late, have better adaptability and flexibility, and usually do not have the inertia of hard to return”* in line 515-517 ( line 456- 459);

*“Chinese officials are trying to avoid corporate fraud, but due to the characteristics of emerging markets in the transition period, Chinese enterprises still face the situation of senior executives cheating in the implementation of equity incentive, which is just like the situation in the western capital markets in the past”* in line 519-522 ( line 460-464);

*“undertake many social functions and the senior executives‘ special status,”* in line 527 ( line 469);

*“The Chinese economy has experienced rapid growth and development these years, however, the system designed for corporate governance lags behind.”* in line 529-531 ( line 471-473);

*“some Chinese scholars believe that executives with relatively short incentive and constraint time may prefer earnings management, stock price manipulation and other methods to meet the exercise conditions; meanwhile, for a long time of incentive and constraint, senior executives are more likely to truly create enterprise value locally by improving operating efficiency, and is also close to the research results on American enterprises, for example, scholars select 1932 firms as sample and reveal that long-term incentives have a stronger inﬂuence on innovation”* in line 538-544 ( line 479-486);

*“The managers have incentives to overinvest in short-term projects to boost the board’s attitude about their abilities, especially”* in line 546-547 ( line 489-490);

*“just as we know that the US capital market is more perfect than the UK, hence, the US management has a lower level of ownership than their British counterparts, which could strengthen the positive effect between equity incentive and corporate performance”* in line 555-558 ( line 497-500);

*“For example, the power of senior executives can ensure the self-serving behavior of senior executives. The greater the power of senior executives, the greater the intensity of equity incentive; Meanwhile, the announcement of the draft equity incentive will produce a significantly positive market response, and the market can be aware of the self-serving behavior of senior executives.”* in line 559-562 ( line 501-506);

*“the study of S&P 500 ﬁrms in the1990s suggests the market value of the company is improved when the pay for performance plan directly for the company's executives is disclosed, the effect of implementing this plan is obvious in companies with potential agency, because this stock compensation mechanism has stimulated the efforts of executives and led to the improvement of company performance.”* in line 566-570 ( line 509-513);

*“On the contrary, the sample of S&P companies in European and American capital markets is more mature, which means more reasonable policy supervision and market expectations”* in line 572-574 ( line 515-517);

*“In spite of the aforementioned theoretical significance and practical enlightenment, however, there are still some imperfections in this paper.”* in line 629-631 ( line 575-577).

**8.The language of the manuscript: the language of the manuscript needs a "Careful Editing" by a native speaker (academic writing style should deserve more attention).**

**Response:** As suggested, we have looked MDPI company for help, they arranged for a senior editor to deal with language problems.

**Additional comments: the author(s) are recommended to do the following to increase the manuscript readability.**

**1. To start with, the manuscript should more clearly and more explicitly spell out its objectives.**

**Response:** As suggested, we have revised the “instruction” in line 82-97 ( line 83-96):

*“As an emerging economy with immature capital market and weak corporate governance, how listed companies to design their equity incentive plans would be more helpful to improving operational performance? Answering this question Therefore, exploring the stock incentive of Chinese listed companies will help to further clarify the economic effect of equity incentive, so as to provide more specific reference value for public firms in emerging market countries.*

*Therefore, Based on the above considerations, this paper study focuses on the impact of design factors such as equity incentive mode, effective period, and other endogenous conditions on the performance of plan implementation and proposes suggestions on the design of equity incentive plans based on the Chinese listed companies. The results of data analysis of 1695 equity incentive cases of China’s listed companies indicate that equity incentive plans are more popular in high-tech industries. Validity periods of incentive plans have significant positive impact on firms’ operational performance, while incentive intensity has a significant negative effect on firms’ operation performance. However, exercise constraints of incentive plans have no significant impact on firms’ operational performance. Our findings have enriched the literature of equity incentive and provide some practical implication for the design of equity incentive plans.”*

**2. Please keep your writing in a scientific writing style, the most effective scientific writing aims for accuracy, clarity and objectivity.**

**Response:** As suggested, we have made language editing, in addition, we also revised the instruction, main body, especially the conclusion part.

**3. Please be careful in using the abbreviations, abbreviated words, and definitions throughout the manuscript. Please see p. 16 table 1 the author(s) defined GROW as follows:**

•	(Profit of this year – Profit of last year) / Profit of last year

I think "GROW" can be defined as follows:

•	Net profit growth (%) = (current period NP-Prior Period NP)/Prior Period NP

**Response:** we have revised in page 17 (table 1) line 328 (line 319)

“Net profit growth (%) = (Current period NP – Prior period NP) / Prior period NP”

**4. Please be careful in using "Punctuating", e.g. (comma, full stop, etc…)** **t****hroughout the paper.**

**Response:** As suggested, we have made several changes throughout the paper, together with English language edit.

**5.Some of the terminology used in the manuscript needs to be unified. For instance, the use of 4 different words that have the same meaning interchangeably could confuse some readers such as "paper/ article/research/study".**

**Response:** As suggested, we have made several changes throughout the paper. Such as line 18, line 63, line 87, line 118, line 137, line 149, line 159, line 172……, and so on.
